# Unification of aggregate growth models by emergence from cellular and intracellular mechanisms

**DOI:** 10.1098/rsos.192148

**Published:** 2020-08-12

**Authors:** T. J. Sego, James A. Glazier, Andres Tovar

**Affiliations:** 1 Department of Intelligent Systems Engineering, Indiana University, Bloomington, IN, USA; 2 Department of Mechanical and Energy Engineering, Indiana University–Purdue University Indianapolis, Indianapolis, IN, USA

**Keywords:** aggregate growth, hybrid cellular Potts model, complex system, emergence

## Abstract

Multicellular aggregate growth is regulated by nutrient availability and removal of metabolites, but the specifics of growth dynamics are dependent on cell type and environment. Classical models of growth are based on differential equations. While in some cases these classical models match experimental observations, they can only predict growth of a limited number of cell types and so can only be selectively applied. Currently, no classical model provides a general mathematical representation of growth for any cell type and environment. This discrepancy limits their range of applications, which a general modelling framework can enhance. In this work, a hybrid cellular Potts model is used to explain the discrepancy between classical models as emergent behaviours from the same mathematical system. Intracellular processes are described using probability distributions of local chemical conditions for proliferation and death and simulated. By fitting simulation results to a generalization of the classical models, their emergence is demonstrated. Parameter variations elucidate how aggregate growth may behave like one classical growth model or another. Three classical growth model fits were tested, and emergence of the Gompertz equation was demonstrated. Effects of shape changes are demonstrated, which are significant for final aggregate size and growth rate, and occur stochastically.

## 1. Background

The growth of cell spheroids and tumours exhibits similar behaviours to ontogenetic growth of various animals [1,2], where growth is regulated by competition for nutrient availability provided by environmental conditions. As a spheroid grows, nutrient levels within the spheroid decrease, while waste products accumulate [3]. Without some intervention, the core of the spheroid experiences an ischaemic condition, which leads to a change in spheroid behaviour, depending on the cell type of the spheroid [4]. For some cell types, like mammary carcinoma cells, ischaemia leads to massive cell death and produces the so-called necrotic core, an inner region with no oxygen [5], minimal living cells and mostly cellular debris [6]. In this case, increased spheroid size and the onset of ischaemia are also believed to induce significant outward diffusion of chemical species (e.g. lactate) that inhibit proliferation at the surface of the spheroid, the total effects of which produce emergent spheroid growth that can be approximated by classical growth models [3]. However, other cell phenotypes like mesenchymal stem cells avoid the formation of a necrotic core altogether by adjusting their metabolism, packing density and secretion of extracellular matrix in response to decreasing available nutrients [7].

The specificity of aggregate growth to cell phenotype and environmental conditions is probably no more evident than in cancer progression, where *in vivo* rates of tumour growth can drastically vary, with potential doubling times on scales as small as a few days to as large as months, depending on the cancer type [8]. A number of classical growth models based on differential equations have been developed to study these considerations and predict aggregate behaviours [9]. These classical models neglect structural heterogeneity and the effects of individual cells, represent total growth as being determined by dynamic relationships between spheroid size, growth rate and environmental conditions, and have been successfully fitted to growth data of specific cell phenotypes [3,10]. Though it has been argued that spheroid growth could follow a ‘universal’ law of this class, where the same mathematical expression, when properly scaled, can describe the growth of solid spheroids [2], no known classical model can successfully predict growth of all phenotypes. To this effect, some classical growth models, namely the Bertalanffy, Gompertz and logistic models, were recently generalized as submodels of a general form, called the unified Richards model, where each of the three submodels can be identified by a single shape parameter of a general mathematical form [11]. The unified Richards model presents a form by which growth data of interest for various cell type can be consistently compared, including maximum relative growth rate, times at maximum growth rate, relative size at maximum growth rate, and initial size [12].

While the classical models, including the recently developed unified Richards model, can provide insights into meaningful biological inquiries and medical considerations in certain scenarios (e.g. for drug discovery) their general usefulness in biomedical applications is limited. Best-fit models of growth data from mammography screenings predicted that *in vivo* tumour growth can be unbounded, potentially compromising the applicability of many classical models like the Gompertz and logistic models for diagnosis [13]. This has been further demonstrated when observing metastasis in mice, where complex mathematical systems, rather than classical growth models, are required to predict the behaviours of aggressive cancer cell types [14]. Furthermore, tumours of high eccentricity are more likely to have aggressive characteristics, demonstrating the significance of shape changes when predicting growth [15], which classical models do not consider. Predictions using limited availability of screening data showed inconsistent accuracy among classical models, where breast and liver cancers exhibited exponential growth, while two neurological cancers (neurinoma and meningioma) followed predictions like those of the Bertalanffy model [16].

As such, mathematical modelling and simulation of aggregate growth have recently shifted attention towards understanding growth dynamics as a complex process [17]. In this paradigm, aggregate behaviour emerges from the interactions of multiple underlying cellular and subcellular mechanisms. Modelling work now places emphasis on cellular-level and multiscale approaches [18], which can capture the effects of intracellular processes and cell motility using computational methods [19]. Most closely to classical models, analytic descriptions of chemical systems and dynamic cell populations have been developed, with varying degrees of complex interactions and predictive capabilities. Analytic models of oxygen diffusion-limited growth could be calibrated to growth data of cerebral organoids when a necrotic core and aggregate shape were assumed [20]. Modelling nutrient diffusion and cell motility as a system of partial differential equations enabled the testing of classical growth models against imaging data of cancer metastasis in mice, and demonstrated the necessity for modelling dynamic aggregate shape [14].

Cellular-level models have the capability to study the emergence of aggregate behaviours and properties in terms of cellular and subcellular processes while considering shape changes and stochasticity. A number of approaches have been developed to predict various aspects of growth dynamics, including hybrid stochastic methods of systems limited by nutrient diffusion [21–25], the utilization of Voronoi tesselations to simulate space-limited mitosis [26], and the effects of metabolites and extracellular matrix production [27]. Among these, the cellular Potts model (CPM) is a widely used discrete kinetic Monte Carlo method that treats individual cells as homogeneous, autonomous agents occupying multiple sites in a lattice and stochastically exchanging lattice sites according to (virtual) energy minimization [28]. The CPM, which is the mathematical framework of cellular dynamics in the commonly used and freely available *Compucell3D* simulation framework [29], can be readily coupled to continuous systems in a hybrid modelling approach like partial differential equations modelling nutrient diffusion and metabolic activity. The CPM is an excellent choice to study how cellular behaviour affects emergent aggregate behaviour compared to other cellular-level agent-based and cellular automata models, because it models deformable cell shape and local cell adhesion, and because it broadly supports model development with suitable hybridization [30].

Using *Compucell3D* or some other implementation, the hybrid CPM (or also the Graner–Glazier–Hogeweg model) has been employed to study a number of factors affecting growth dynamics of aggregates. Simulations of avascular tumours using the CPM and while assuming (but not simulating) a single diffusive nutrient produced expected characteristics of diffusion-limited growth curves, including early exponential, later linear and final asymptotic growth [31]. Simulation of multiple chemical species regulating cell proliferation and viability produced growth curves that could be fitted with a Gompertz equation [32]. *In vivo* scenarios have been constructed in virtual tissue models to study factors affecting cancer behaviour and invasiveness, including environmental fibre density and orientation of the extracellular matrix [33], the Wnt distribution of the colorectal crypt [34], and interactions with a local vasculature [35]. More generally, individual cellular mechanisms have been simulated to study their effects on emergent behaviour, including mechanisms affecting aggregate interface morphology and the origin of global shape changes during growth [36], regulation of cell proliferation during growth by cell compressibility and contact inhibition [37] or cell deformation [38], and space-limited mitosis and resulting logistic model behaviour during monolayer growth [39]. Simulations of scenarios in scaffold-free biofabrication have demonstrated the effects of insufficient oxygenation on oversized spheroids and the potential for environmental control during *in vitro* fabrication of tissue [40].

Among the aforementioned contributions, there remains a sufficient explanation of the seemingly selective accuracy of the classical growth models when modelling diffusion-limited multicellular aggregate growth. Plainly stated, why do some classical models work well for aggregate growth of some cell types, and other models for other types? A hybrid CPM is well suited to interrogate this phenomenon for several reasons: (i) it permits global shape changes, (ii) it allows modelling according to cell phenotype specificity, (iii) it supports the simulation of an arbitrary number of diffusive chemical species, (iv) it considers volume exclusion, and (v) it permits analysis of cellular and intracellular mechanisms that are directly relatable to other modelling works and developed virtual tissue simulations. A sufficient explanation of the disagreement among the classical models using a robust framework like a hybrid CPM would provide a better understanding of the responsible mechanisms, as well as enhanced modelling and predictive capabilities. Better simulation environments could then be constructed concerning the interactions of diffusive chemicals and cell viability, for medical applications like developing scalable production of tumour spheroids for drug discovery [41] and computational tools to aid cancer prognosis using patient-specific imaging [42], to the development and application of bioreactors in biofabrication [43,44].

In this work, a hybrid CPM was developed and employed to explain the disagreement among the classical models of diffusion-limited growth *in silico* as emergent behaviours from the same multiscale model system. This was accomplished by representing the intracellular processes associated with cell proliferation and death as stochastic functions of internal chemical conditions using Gaussian distributions [40] for a single diffusive nutrient provided by the environment. In this paradigm, parameters of the stochastic functions represent phenotype-specific genetic regulation by which each cell responds to its local oxygen such that a set of model parameter values is an identifier of a specific cell phenotype. Emergence of classical growth models was demonstrated by fitting the unified Richards model to two-dimensional simulation results, where also the Bertalanffy, Gompertz and logistic growth models were tested. Parameter variations of the intracellular model were performed to show that an emergent growth curve may behave more or less like a particular classical growth model depending on the type-specific parameters of the same model system, which was accomplished by comparing unified Richards model shape parameters of fitted results, as well as goodness of fit to simulation results using the unified Richards and classical growth models. Shape analysis demonstrated the necessity of considering shape changes when simulating multicellular aggregate growth.

## 2. Methods

Cellular dynamics is simulated using the CPM for a regular, two-dimensional lattice L of cells in a medium, henceforth collectively referred to as agents. Each *i*th agent is initialized with a unique identification integer $\sigma_{i}\in I\subset N$ such that, for the discrete coordinate r∈L, the virtual time k∈N and σ : L→I, lattice sites occupied by the *i*th agent are denoted *σ*(*r*, *k*) = *σ*_*i*_. L is mapped to a physical, continuous domain P by a modelling coefficient α : L→P such that x=αr∈P is a physical coordinate corresponding to the discrete coordinate *r*. Every agent is modelled with a set of modelling descriptors according to its type $\tau_{i}=\tau (\sigma_{i})\in T\subset N$, where two cells of the same type are modelled identically and the medium is assigned an integer value of zero for identification and type.

Cell motility is simulated by considering a set of randomly selected lattice events called copy attempts, called a Monte Carlo step (MCS), where one MCS signifies having accomplished a number of copy attempts equal to the number of lattice sites. For each copy attempt in a MCS, a source site $r^{s}\in L$ and target site $r^{t}\in N^{1}(r^{s})$ in the von Neumann neighbourhood of range one $N^{1}\subset L$ of the source site are both randomly selected. For the *k*th MCS, if the copy attempt is accepted then, at virtual time *k*′ > *k* after the copy attempt (i.e. *k* < *k*′ ≤ *k* + 1), *σ*(*r*^*t*^, *k*′) = *σ*(*r*^*s*^, *k*). The probability of each copy attempt is considered as the stochastic rule,

2.1 $$\mathrm{Pr}(\sigma (r^{t},k^{\prime })=\sigma (r^{s},k))=\exp\left(-\max\left\{0,\frac{\Delta H}{H^{*}}\right\}\right),$$

where ΔH is the change in the system effective energy H due to the copy attempt and $H^{*}=10$ is the intrinsic random motility. In this way, copy attempts are accepted such that the system effective energy tends to decrease and the likelihood of an increase in effective energy decreases proportionally to the magnitude of the increase. The system effective energy H consists of effective energies independently associated with cell size and interface conditions. A spatial constraint $v^{\tau }\in R_{+}$ is imposed on each cell such that the space $\alpha^{2}∥L^{\sigma_{i}}∥$ of the subdomain $L^{\sigma_{i}}=\{r\in L\;:\;\sigma (r,k)=\sigma_{i}\}$ occupied by cell *σ*_*i*_ tends towards $v^{\tau_{i}}$. A modelling coefficient $J_{\tau ,\tau^{\prime }}=J_{\tau^{\prime },\tau }\in R_{+}$ describes the interface condition between agent types *τ* and *τ*′. For both the spatial constraints and interface conditions,

2.2 $$H=\sum_{\sigma_{i}}\lambda_{v}^{\tau_{i}}{\left(∥L^{\sigma_{i}}∥-v^{\tau_{i}}\right)}^{2}+\sum_{r}\sum_{r_{i}\in N(r)}(1-\delta_{\sigma (r,k),\sigma (r_{i},k)})\;J_{\tau (\sigma (r,k)),\tau (\sigma (r_{i},k))},$$

where the spatial Lagrange multiplier $\lambda_{v}^{\tau }$ is a modelling coefficient for cell type *τ* and the medium has no spatial constraint (i.e. $\lambda_{v}^{0}=0$, $\lambda_{v}^{\tau }=2$ otherwise), N(r)⊂L is the neighbourhood of *r*, *δ*_*p*,*q*_ is the Kronecker delta. For all simulations in this work (but not necessarily in all CPM applications), N corresponds to the Moore neighbourhood of range one. All simulations in this work are performed for a single, theoretical phenotype of nominal diameter 20 μm (i.e. $v^{\tau_{i}}=314\;\text{μ}{\text{m}}^{2}$), and the length *α* of each site is 2.96 μm so that a nominal cell occupied 36 lattice sites when initialized as a square, which is large enough to prevent accidental cell death due to cells being too small during splitting. Interface coefficients are selected such that the aggregate tended to stay round by minimization of contact energy, with cell–cell *J*_1,1_ = 6 and cell–medium *J*_0,1_ = 12. As such, adhesion parameters in the regime of *J*_0,1_ > *J*_1,1_ affect the tendency of aggregate shape changes, but otherwise do not affect the dynamics of aggregate growth.

The modelling of diffusive oxygen in P is performed by solving a parabolic partial differential equation. For oxygen concentration *c* = *c*(*x*, *t*), homogeneous diffusivity *D* = 2500 μm^2^ [20] and source field *s* = *s*(*x*, *t*) = −0.5 fmol/cell s^−1^ [20],

2.3 $$\frac{\partial c}{\partial t}=\sum_{j}D\frac{\partial^{2}c}{\partial x_{j}^{2}}+s,$$

where *x*_*i*_ ∈ *x* is along the *i*th dimension of P, *s* maps respiration in L onto the rate of oxygen and *t* = *βk* is the physical time related to simulation step *k* by the modelling coefficient *β* = 0.1 s/MCS [40], which was chosen to be sufficiently large so that chemical diffusion occurred much faster than cellular motility. The system is solved using a second-order central difference scheme of P, forward Euler explicit integration of time and a prescribed boundary condition that all medium sites remain at an environmental concentration of 0.2 mM [20,32]. A sufficiently high, but otherwise arbitrary, value was selected for the time correlation *β* such that transient effects of cell motility were negligible.

The phenotypic responses of proliferation and death for cell *σ*_*i*_ are modelled as stochastic functions of the mean concentration field ${\bar{c}}^{\sigma_{i}}(t)$ in its physical subdomain $P^{\sigma_{i}}=\{x\in P\;:\;\sigma (\alpha^{-1}x,\beta^{-1}t)=\sigma_{i}\}$ (i.e. $P^{\sigma_{i}}=\alpha L^{\sigma_{i}})$. The probability of a *j*th phenotypic response $\tau_{i}\to \tau_{j}^{\tau_{i}}$ due to oxygen is then considered once every time step for each cell according to a normal distribution,

2.4 $$\mathrm{Pr}(\tau_{i}\to \tau_{j}^{\tau_{i}})=\beta p_{j}^{\tau_{i}}\exp\left(-{\left(\frac{{\bar{c}}^{\sigma_{i}}-c_{j}^{\tau_{i}}}{\gamma_{j}^{\tau_{i}}}\right)}^{2}\right),$$

where the probability rate $p_{j}^{\tau_{i}}$, standard deviation $\gamma_{j}^{\tau_{i}}$ and target $c_{j}^{\tau_{i}}$ are modelling coefficients for the cell type *τ*_*i*_. For every *τ*, there is a corresponding dead *τ*-type, where metabolic activity ceases and a spatial constraint of zero is assigned so that, over time, dead cells tend to be removed from the simulation. For cell splitting, a cell is divided along its minor axis, from which a new agent is initialized and added to the simulation.

Aggregate area was measured in intervals of 1k MCS (denoted ‘kMCS’) and fitted to the unified Richards (or U-Richards) model form [11,12],

2.5 $$W(k)=A{\left(1+\left({\left(\frac{W_{0}}{A}\right)}^{1-S}\;-\;1\right)\exp\left(-\frac{Gk}{S^{S/(1-S)}}\right)\right)}^{1/(1-S)},$$

where *A* is the upper asymptote of the curve, *W*_0_ = *W*(0), *G* is the growth rate and *S* is a shape parameter. Note that the Bertalanffy, Gompertz and logistic models can all be derived by *S* = 2/3, *S* → 1 and *S* = 2, respectively. In the case of the Gompertz model, equation (2.5) takes the form

2.6 $$\lim_{S\to 1}W(k)=A{\left(\frac{W_{0}}{A}\right)}^{\exp(-eGk)}.$$

For all experimental fits, results were fitted by normalizing simulation time to the interval [0, 1], and fitted growth rates were scaled to units of 100 kMCSs^−1^. Results were fitted to the unified Richards, Bertalanffy, Gompertz and logistic models, the results of which were compared.

To generalize the effects of various intracellular response profiles for proliferation and death in a biologically meaningful way, the probability rate and standard deviation in equation (2.4) were cast in terms of specified concentration response widths and population multipliers according to exponential growth. Response width *w*_*j*_ is measured as the absolute difference in target and cut-off concentrations $c_{j}^{\tau_{i}}$ and $c_{j}^{\tau_{i},o}$, respectively, of the *j*th response. For each *j*th response, the corresponding probability rate $p_{j}^{\tau_{i}}$ was calculated according to a population multiplier *n*_*j*_ with respect to 10 kMCSs, where for *N*(*k*) cells at virtual time *k*, population multiplier *n* and rate *p*, a general form was employed,

2.7 $$n(k)=\frac{N(k)}{N(0)}={\left(1+p\right)}^{k}.$$

A probability rate $p_{j}^{\tau_{i},o}$ at the cut-off concentration was set to occur with a probability of once in one million MCSs. Then for cut-off concentration $c_{j}^{\tau_{i},o}$ and corresponding probability rate $p_{j}^{\tau_{i},o}$, all profiles of interest where calculated according to parametrizations of equation (2.4) using equation (2.7),

2.8 $$p_{j}^{\tau_{i}}=n_{j}^{10\;000^{-1}}-1$$

and

2.9 $$\gamma_{j}^{\tau_{i}}=\frac{w}{\sqrt{\ln p_{j}^{\tau_{i}}-\ln p_{j}^{\tau_{i},o}}}.$$

**Figure 1.**
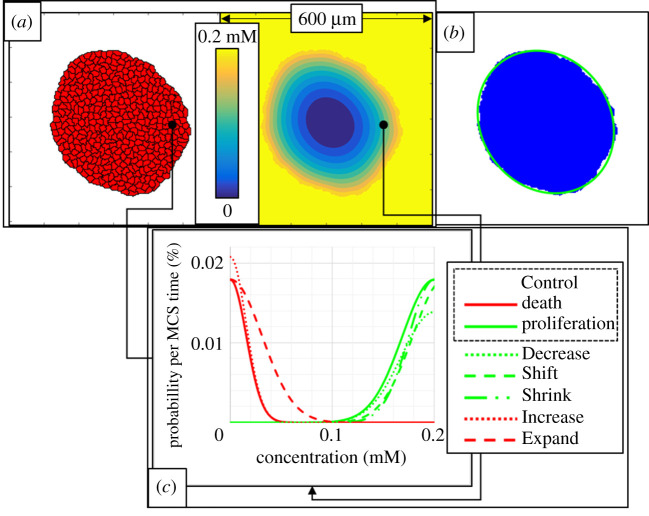
Summary of growth dynamics simulation and analysis. (*a*) Discrete domain of cells (left) and corresponding continuous domain of oxygen (right). (*b*) detected aggregate domain (blue) and best-fit ellipse (green). (*c*) Intracellular model profiles for the first set of variations. Red and green curves correspond to probability rates of cell death and proliferation, respectively, as a function of the mean oxygen concentration.

Variations in the intracellular modelling coefficients were considered for both proliferation and death with respect to an initial total profile, labelled ‘Control’. Variations were performed for the target concentration of proliferation, and the response width and rate of both proliferation and death. Control was selected such that, for the experimental set-up of five initial cells, aggregate growth converged to a final size that could be contained within a square domain of 600 μm width, and well before 100 kMCSs. To simplify computational experiments, all variations were selected to reduce aggregate final size. All profiles were simulated until a U-Richards fit well captured aggregate final size, and for at least 100 kMCSs. Profiles were named according to their corresponding variation, which are designed to characterize the ways in which two cell types may differ in their response to local oxygen according to the parameters of the model. They are, Decrease, for a decrease in proliferation rate (a cell type that proliferates less aggressively); Shift, for an upward shift in proliferation target (a cell type that nominally proliferates in a higher oxygen concentration); Shrink, for a decrease in proliferation response width (a cell type that proliferates more selectively according to local oxygen); Increase, for an increase in death rate (a cell type that dies more rapidly when hypoxic); and Expand, for an increase in death response width (a cell type that dies over a greater range of low oxygen concentrations, figure 1). Two sets of these variations were performed and fitted to equation (2.5). The first set was plotted to show the effects of individual intracellular parameters on emergent growth curves, while emergent coefficients from the Control profile and two variations of an intracellular coefficients were tested for linearity. All simulations, data and shape analysis, and curve fitting were performed in Matlab (The MathWorks, Inc., Natick, MA, USA). Simulations consisting of 100 kMCSs typically required approximately 12 h to complete. Measurements of eccentricity were made using Matlab Image Processing Toolbox.

## 3. Results

**Figure 2.**
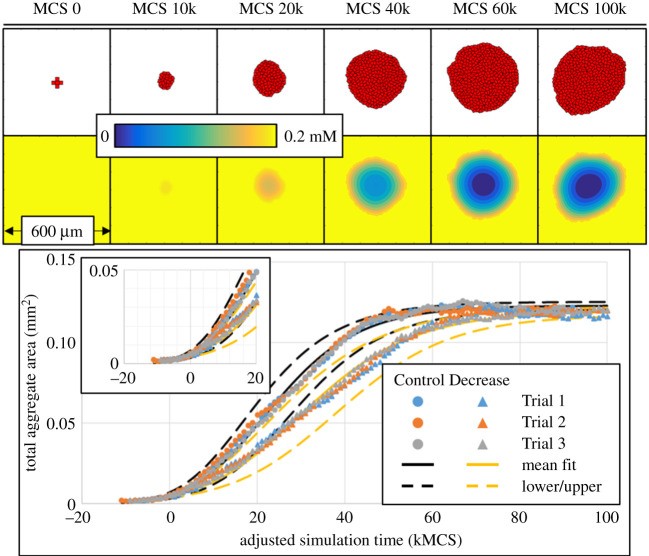
Growth dynamics and saturation *in silico*. Cellular configuration (top) and corresponding oxygen field (middle) for a trial using the Control intracellular profile at various simulation times. Bottom: results from three trials using control and decrease profiles, with fits using the unified Richards model. Lower/upper fits calculated as rightmost/leftmost curves considering all fitted parameter means and three standard deviations.

**Figure 3.**
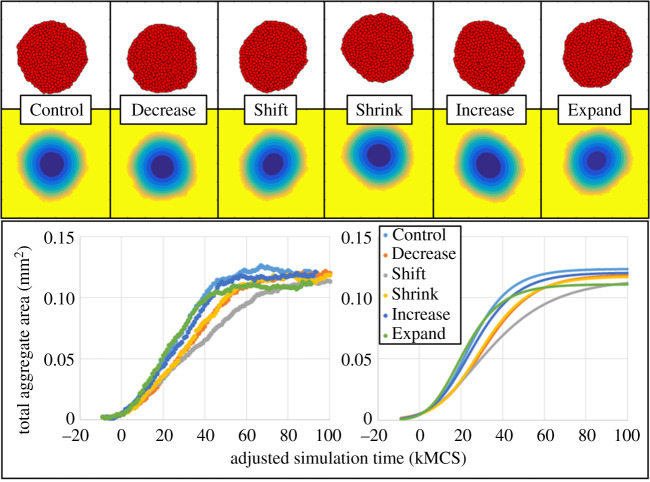
Resulting growth dynamics for the first set of variations in individual intracellular parameters. Resulting configurations at MCS 100k (top), with corresponding oxygen field (middle) and normalized probability distribution for proliferation and death (bottom). Oxygen contours are scaled the same as in figure 2. Proliferation and death probabilities are shown as red and green, respectively, and are normalized by the probability rate ($p_{j}^{\tau_{i}}$) of the corresponding intracellular model profile. Bottom: total aggregate area data (left, dots) and fits (right, lines) versus adjusted stimulation time using the unified Richards model. Shown results and fits for Control and Decrease are per trials with best coefficient of determination.

Three trials of the Control and Decrease 1 parameter sets were simulated and fitted to the U-Richards model, to interrogate the variation of growth dynamics metrics for a given intracellular parameter set (figure 2). Trials using the Control parameter set were simulated for 100 kMCSs, while trails for the Decrease 1 parameter set were simulated for 150 kMCSs to ensure a steady-state final size. All six trials were observed to well fit the U-Richards model, though growth dynamics metrics did show some variation among the trials of each intracellular parameter set when fitted to a U-Richards model with an initial cell number of five. This is probably due to a non-negligible degree of variation presented by sampling the probability of proliferation for only five cells at a time, which, though approximately constant due to minimal oxygen gradients generated by so few cells, can exhibit significant variations in the amount of time required to observe a significant number of proliferative events. However, analysis of results showed that this effect could be mitigated by adjusting the simulation time at which the U-Richards model considers a result to be an initial measurement (i.e. the parameter *W*_0_ of equation (2.5)) while still considering significant aggregate growth. In the present case, an initial measurement of 15 cells was found to generate very similar growth dynamics metrics among each trial of both parameter sets (at kMCS 8, 11 and 7 for Control trials, and at kMCS 9, 8 and 9 for Decrease 1 trials). Because of this, an adjusted simulation time was calculated from the first observed result with 15 cells and used for fitting results to all growth models of interest (note the horizontal shift in results of figures 2, 3 and 5 such that very early results are considered as negative adjusted simulation time). The simulation at which the adjusted simulation time is zero (i.e. Initial time) is listed for each trial in table 2.

Having accounted for initial effects, simulation results for trials using each parameter set were noticeably similar when measured by adjusted simulation time (figure 2). Among U-Richards fits for trials of each parameter set, the mean and standard deviation of U-Richards coefficients were calculated, and all standard deviations were less than 13% of their respective mean (from equation (2.5), Control: *A* = 123.1 ± 0.7472 μm^2^, *W*_0_ = 5.143 ± 0.7472 μm^2^, *G* = 2.687 ± 0.05094/100 kMCSs, *S* = 1.393 ± 0.03815; Decrease 1: *A* = 119.8 ± 0.8452 μm^2^, *W*_0_ = 4.758 ± 0.3434 μm^2^, *G* = 2.127 ± 0.03741/100 kMCSs, *S* = 1.504 ± 0.08467). U-Richards curves were generated and compared to simulation results, where the mean U-Richards coefficients generated curves that matched very well with results (i.e. the curves shown as solid lines in figure 2), and rightmost and leftmost curves were generated from coefficients three standard deviations greater than and less than their respective means (i.e. the curves shown as dashed lines in figure 2). By inspection, these rightmost and leftmost curves enclosed nearly all deviations of simulation results from their respective mean U-Richards curves, which were observed to occur most significantly before an adjusted simulation time of 20 kMCSs. Given the similarity of measured U-Richards coefficients among all trials of each parameter set and neglecting significant aggregate shape changes, results for individual trials are then probably representative of the emergent dynamics of a parameter set, despite all of the underlying stochasticity.

For both parameter sets, the mean shape parameter *S* from equation (2.5) implied no classical growth model (from table 2, mean *S* = 1.393 for the Control set, and mean *S* = 1.504 for the Decrease 1 set), but was instead well between the Gompertz (i.e. *S* → 1, equation (2.6)) and logistic (i.e. *S* = 2) growth models. Results from the Control set were closer to a Gompertz model curve than those of Decrease 1, the mean shape parameter of which was approximately equidistant from those of the Gompertz and logistic models. The decrease in proliferation rate from the Control set to Decrease 1 did not significantly decrease the upper asymptotic aggregate size (2.702% decrease in mean U-Richards upper asymptote), while the effect was clearly observed by a 20.83% decrease in the fitted U-Richards growth rate. The results then imply that, using the intracellular model (equation (2.4)), a decrease in the probability rate of proliferation alone more significantly decreases the rate at which an aggregate will reach its final size, rather than its final size *per se*. Considering final size as the equilibration of cell proliferation and death rates, one would expect that, along with a decrease in aggregate growth rate, a significant decrease in proliferation rate would result in a significant decrease in final size. Results do not support this paradigm for the tested decrease in proliferation rate. Quantitatively, the tested decrease in proliferation rate constitutes a 33% decrease in population at nominal oxygen values and 10 kMCSs according to exponential growth (by equation (2.8)), and yet only a mean decrease in final aggregate size of less than 3%, and a 3.693% decrease among the extrema (table 2).

The remaining first set of variations in intracellular parameters was then simulated and fitted to the U-Richards model for all aforementioned variations of interest (figure 3). For comparison of the effects of variations in individual intracellular parameters, the trial with the best fit to the U-Richards model was selected from each set of trials (Trial 3 from figure 2 for both Control and Decrease, with *R*^2^ values of 0.9961 and 0.9976, respectively). Growth model fits of results from various intracellular parameters were henceforth compared to this selected Control trial. Like Decrease 1, the intracellular parameters for Shift 1 and Shrink 1 were also simulated to 150 kMCSs, while the simulation for the Decrease 1 parameters reached its final size at nearly the same simulation time as that of the Control parameters, and as such was simulated for 100 kMCSs.

For all simulated intracellular parameter sets in this first set of variations in model parameters of equation (2.4), the worst coefficient of determination *R*^2^ for a U-Richards model fit was 0.9940 (Increase 1, table 2). All trials were also fitted to the classical growth models. As expected, the U-Richards model produced the best fit for each trial, due to its additional degree of freedom (i.e. it has one more coefficient to fit than the classical models). The coefficient of determination for each classical growth model fit showed a clear correlation with the U-Richards model shape parameter (e.g. a shape parameter near 1 correlated with a best fit to the Gompertz model among the three classical growth models that were tested, while a shape parameter farther from 2 showed a coefficient of determination farther from 1 for the logistic model, table 2). As such, the tendency of a phenotype to behave collectively like a particular classical model (or like no particular classical model) can be measured by the U-Richards model shape parameter. Upper asymptote was marginally affected by an increase in death rate (Increase 1, 2.459% decrease in *A* from equation (2.5)), and most affected by an expansion of the death response width (Expand 1, 10.17% decrease in *A*), while an upward shift in proliferation target most significantly decreased the fitted U-Richards growth rate (Shift 1, 37.20% decrease in *G* from equation (2.5)). Marginal decrease in upper asymptote for Decrease 1 could at least be partially explained by an observed eccentricity in aggregate shape at 100 kMCSs, which was also observed in results for Shift 1 at 100 kMCSs, since fewer cells in the interior of the aggregate are exposed to hypoxic conditions (consider if the morphology were instead a plane). Interestingly, an expansion of the death response width slightly increased the fitted U-Richards growth rate (Expand 1, 6.799% increase in *G*), which was probably at least partially a consequence of the stochasticity of the system, since the U-Richards fit *R*^2^ was 0.9969. The U-Richards shape parameter was nearly unaffected for an increase in death rate (Decrease 1, 0.5673% decrease in *S* from equation (2.5)) and marginally affected by a decrease in proliferation response width (Shrink 1, 6.541% increase in *S*), while a shift towards a Gompertz growth model was observed for an expansion of the death response width (Expand 1, 11.25% decrease in *S*). Probably most significantly from these simulation results, a positive shift in proliferation response nearly produced results that fit a U-Richards model (Shift 1, 25.68% decrease in *S*, *R*^2^ = 0.9959) with a shape parameter 95% confidence interval (*S* = 1.063 ± 0.06899) containing the limit for which the fit is that of the Gompertz growth model (i.e. *S* → 1).

**Figure 4.**
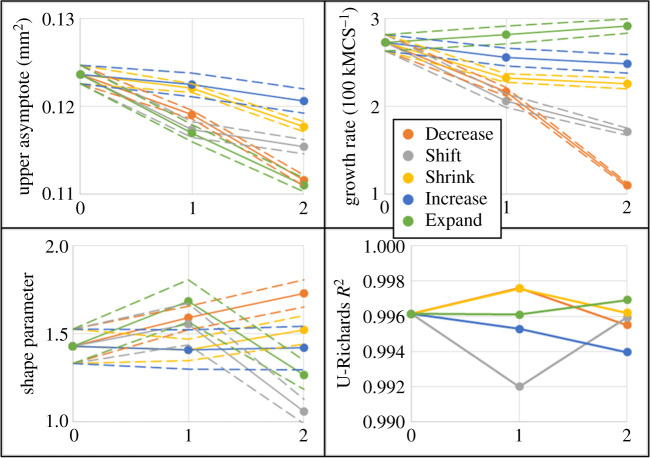
Test for linearity experiment. 95% confidence intervals shown as dashed lines. Horizontal axes annotate Control as ‘0’, and ‘1’ and ‘2’ as midpoint and endpoint variations with respect to intracellular model parameters of interest according to table 1.

To test the predictability of U-Richards growth model parameters with respect to intracellular model parameters, a second set of variations in model parameters of equation (2.4) was simulated to convergence of aggregate size and fitted to the U-Richards growth model, according to table 1. For all intracellular parameters except Decrease 2, a linear variation of parameters was designed by selecting each second variation as the midpoint between the corresponding Control parameter and that of the first set of variations (e.g. the expansion of death response width using equation (2.4) from Control in Expand 2 is half of that of Expand 1). For the case of Decrease 2, the first variation in proliferation rate was designed to be the midpoint, since final aggregate size did not significantly decrease from that of the Control set and final shape did not exhibit significant eccentricity. On the other hand, while final aggregate size from Increase 1 also did not significantly decrease from that of the Control set, it did exhibit significant eccentricity, and so Increase 2 was designed to be a midpoint. According to this scheme, variations are henceforth referred to as midpoint and endpoint variations (figure 4). Like in the first set of parameter variations, Shrink 2 and Shift 2 were simulated for 150 kMCSs, while Decrease 2 was simulated for 250 kMCSs.

**Table 1.** Parameters of all simulated intracellular model profiles according to the biological parametrizations in equations (2.8) and (2.9), from which the model parameters in equation (2.4) were generated. Units of response target and width are micromolar concentration (μM). Variations from Control are italicized.

	proliferation			death		
profile	target (μM)	width (μM)	multiplier	target (μM)	width (μM)	multiplier
Control	200	100	6	0	50	6
Decrease 1	200	100	*4*	0	50	6
Shift 1	*210*	100	6	0	50	6
Shrink 1	200	*80*	6	0	50	6
Increase 1	200	100	6	0	50	*8*
Expand 1	200	100	6	0	*100*	6
Decrease 2	200	100	*2*	0	50	6
Shift 2	*205*	100	6	0	50	6
Shrink 2	200	*90*	6	0	50	6
Increase 2	200	100	6	0	50	*7*
Expand 2	200	100	6	0	*75*	6

For all simulated intracellular parameter sets in this second set of variations, the worst coefficient of determination *R*^2^ for a U-Richards model fit was 0.9920 (Shrink 2, table 2). The fitted U-Richards model upper asymptote behaved fairly predictably for all variations, where all upper asymptotes resulting from midpoint variations were less than that of the Control set, and greater than those of corresponding endpoint variations. Variations in intracellular model parameters listed in table 1 and resulting U-Richards fit coefficients listed in table 2 were tested for linearity by examining goodness of fit when modelling U-Richards fit coefficients as linear functions of individual intracellular model parameters. The response of upper asymptote to a linear increase in death response width was remarkably linear, where a line fit measured upper asymptote to simulated death response widths with *R*^2^ = 0.9986, while all other responses were not especially linear. Likewise, a linear variation in death response width also demonstrated a nearly perfect linear response in U-Richards model growth rate (a line fit measured growth rates to simulated death response widths with *R*^2^ of 0.9995), with all other linear fit *R*^2^ values less than 0.9697. However, the linear variations in death response width exhibited a notably nonlinear response in U-Richards shape parameter, where the midpoint result was much greater than those of Control and endpoint. This contrasts with a strong linear response in U-Richards shape parameter for a linear variation in proliferation rate, which fit to a line with *R*^2^ = 0.9983 (all linear fit *R*^2^ values were less than 0.5867 when fitting shape parameter to all other intracellular model parameters).

**Table 2.** Fit coefficients and metrics of fitted emergent growth curves. Top: coefficients of the unified Richards growth model (equation (2.5)) for all intracellular response profiles, according to table 1. ± values indicate 95% confidence interval half-widths. Bottom: coefficient of determination of fitted growth models of interest.

	unified Richards fit coefficients				
	upper asymptote	initial area	growth rate *G*	shape	initial
trial	*A* (mm^2^)	*W*_0_ (mm^2^)	(100 kMCSs)^−1^	parameter *S*	time (MCSs)
Control	0.1222 ± 0.001195	0.005093	2.703 ± 0.1046	1.394 ± 0.1143	8000
	0.1234 ± 0.001371	0.005766	2.630 ± 0.1075	1.354 ± 0.1245	11 000
	0.1236 ± 0.001030	0.004569	2.728 ± 0.09293	1.430 ± 0.09631	7000
Decrease 1	0.1195 ± 0.0009811	0.004543	2.095 ± 0.08017	1.421 ± 0.1137	9000
	0.1207 ± 0.0007499	0.004578	2.119 ± 0.06377	1.501 ± 0.09068	8000
	0.1191 ± 0.0004992	0.005154	2.168 ± 0.04557	1.591 ± 0.06837	9000
Expand 1	0.1111 ± 0.0007707	0.004447	2.913 ± 0.08141	1.269 ± 0.08180	9000
Increase 1	0.1206 ± 0.001385	0.005067	2.484 ± 0.1061	1.422 ± 0.1238	7000
Shrink 1	0.1177 ± 0.0005813	0.004648	2.262 ± 0.05806	1.523 ± 0.08008	6000
Shift 1	0.1154 ± 0.0008110	0.004744	1.713 ± 0.04079	1.063 ± 0.06899	8000
Decrease 2	0.1116 ± 0.0005863	0.005163	1.107 ± 0.02593	1.730 ± 0.07835	16 000
Expand 2	0.1170 ± 0.0009594	0.005425	2.817 ± 0.1014	1.687 ± 0.1190	9000
Increase 2	0.1225 ± 0.001329	0.004866	2.562 ± 0.1018	1.410 ± 0.1116	11 000
Shrink 2	0.1174 ± 0.0009199	0.004578	2.065 ± 0.07872	1.555 ± 0.1171	6000
Shift 2	0.1221 ± 0.0004837	0.004630	2.325 ± 0.04654	1.409 ± 0.06052	9000

	coefficients of determination *R*^2^			
trial	unified Richards	Bertalanffy	Gompertz	logistic
Control	0.9947	0.9830	0.9915	0.9896
	0.9942	0.9848	0.9918	0.9889
	0.9961	0.9828	0.9922	0.9917
Decrease 1	0.9915	0.9782	0.9876	0.9868
	0.9949	0.9798	0.9899	0.9918
	0.9976	0.9798	0.9909	0.9956
Expand 1	0.9969	0.9883	0.9953	0.9891
Increase 1	0.9940	0.9830	0.9907	0.9899
Shrink 1	0.9962	0.9803	0.9908	0.9934
Shift 1	0.9959	0.9920	0.9958	0.9817
Decrease 2	0.9955	0.9765	0.9872	0.9948
Expand 2	0.9961	0.9766	0.9879	0.9950
Increase 2	0.9953	0.9840	0.9921	0.9909
Shrink 2	0.9920	0.9750	0.9859	0.9895
Shift 2	0.9976	0.9846	0.9940	0.9930

**Figure 5.**
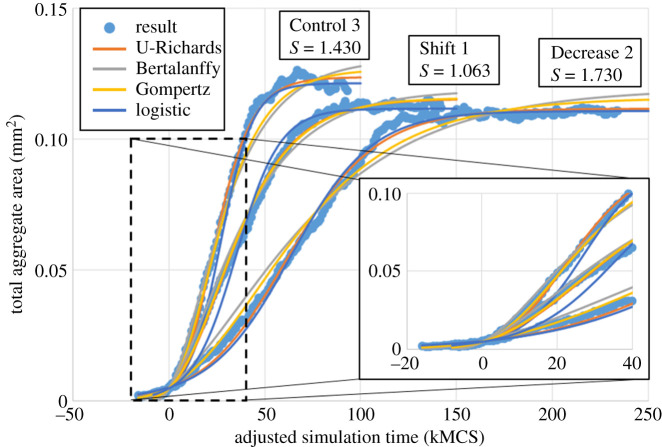
Fit comparison showing emergent Gompertz and nearest logistic equations. Shape parameter *S* shown for each fit using the unified Richards model.

The second set of simulated variations also produced the two greatest fitted U-Richards shape parameters (from equation (2.5), Decrease 2 *S* = 1.730, and Expand 2 *S* = 1.687, table 2), which was closest to a logistic growth model, i.e. *S* = 2. Considering Decrease 2 as producing the most logistic model-like results and, as previously discussed, Shift 1 as producing the most Gompertz model-like results (*S* → 1), simulation results then produced U-Richards model shape parameters that were as near 13.50% to a logistic growth model, and 6.300% to a Gompertz growth model, by using different parameters of the same intracellular model (equation (2.4)). Figure 5 shows fits to these trials and Control 3 using the U-Richards and classical models, where, most notably, the U-Richards and Gompertz model fits are indistinguishable by inspection for Shift 1, and the same is nearly so for the U-Richards and logistic models when applied to Decrease 2.

This was also demonstrated by fitting the Bertalanffy, Gompertz and logistic growth models to all simulation results, the coefficients of determination of which showed the same observation (table 2): results from the Shift 1 parameters best fit the Gompertz model among the three (*R*^2^ = 0.9920, 0.9958, 0.9817 using the Bertalanffy, Gompertz and logistic models, respectively), results from the Decrease 2 parameters best fit the logistic model among the three (*R*^2^ = 0.9765, 0.9872, 0.9948 using the Bertalanffy, Gompertz and logistic models, respectively), and results from the Control parameters produced a U-Richards shape parameter near the midpoint of those of the Gompertz and logistic growth models (*R*^2^ = 0.9828, 0.9922, 0.9917 using the Bertalanffy, Gompertz and logistic models, respectively). Of course, in the case of the Control parameters, the Gompertz and logistic models both would be considered useful by these measures of their fits to results. This, however, is besides the point in that they are both submodels of the U-Richards model, which showed an even better measure of being able to fit to results (*R*^2^ = 0.9961), and in that both submodels may or may not emerge from the intracellular model of the present work (equation (2.4)), depending on the responsiveness of the particular cell phenotype to local chemical conditions.

**Figure 6.**
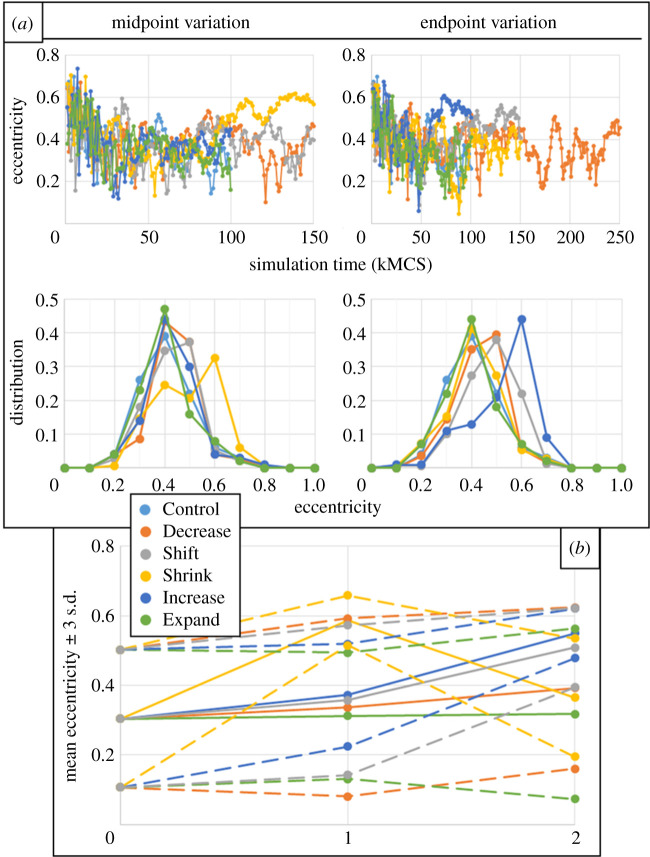
Eccentricity of fitted ellipses for results from all intracellular parameter variations. (*a*) Eccentricity for the midpoint (left) and endpoint (right) parameter variations at simulation time (top) and distribution during simulation (bottom). (*b*) mean measurements (solid lines) plus or minus three standard deviations (dashed lines) of the last 25 kMCSs of simulation for Control (0), midpoint (1) and endpoint (2) variations.

To interrogate the possible causes and effects on growth dynamics of aggregate shape, the eccentricity of aggregate configurations for the Control parameter set and all midpoint and endpoint variations was measured every 1 kMCS using a best-fit ellipse (as demonstrated in figure 1*b*), beginning with MCS 1k (figure 6). The distribution of eccentricity during simulation was approximated by calculating a normalized histogram of all eccentricity measurements with bin intervals of 0.1. The effects of eccentricity on final size was characterized by calculating the mean and standard deviation of eccentricity for the final 25 kMCSs of simulation time. By these measures, aggregates with a consistently eccentric final shape could be identified by measurements with a high mean eccentricity and a low standard deviation, while inconsistent eccentricity could be identified by measurements with a high standard deviation of eccentricity. For the present case, a low eccentricity standard deviation was in the range of 0.02 to 0.04. For reference, the mean of all mean measurements of eccentricity of all trials was 0.3875, and the mean of all standard deviations was 0.1081.

As alluded to by the mean of all mean measurements, the peak of most eccentricity distributions was near 0.4, while only three measurements were as low as near 0.1 (endpoint variations for Shift, Shrink and Increase, figure 6), and two measurements were as high as near 0.8 (midpoint variations for Shrink and Increase). With the exception of variations for Expand, upward shifts in eccentricity distribution were observed for all parameter variations, and especially for the midpoint decrease in proliferation response width and endpoint increase in death rate using equation (2.4). For the case of variations in proliferation response width, i.e. Shrink, the increase in eccentricity for the midpoint variation demonstrated a degree of randomness with which an aggregate can become eccentric, which particularly occurred during the asymptotic stage of aggregate growth (figure 6*a*, top left). This seems to reflect an increase in the corresponding fitted U-Richards upper asymptote (figure 4), where the midpoint variation deviated upward from a linear fit. Eccentricity during the same stage of growth was observed to increase with increasing death rate (i.e. Increase), especially for the endpoint variation, probably then mitigating some of the effects of the variation on fitted U-Richards upper asymptote (supposing that, without the effects of eccentricity, the upper asymptote of figure 4 would have decreased for Increase variations much more than what was observed). No observed behaviour from shape analysis explained the seemingly erratic behaviour of the fitted U-Richards shape parameter shown in figure 4 for variations in proliferation response target and width, suggesting that the fitted shape parameter and growth rate exhibit some coupled response with respect to these intracellular model parameters that could not be detected with the method of this work.

Variations for Expand (i.e. increases in equation (2.4) model response width for cell death) instead produced remarkably similar distributions of eccentricity over all simulation results, both of which were similar to that from the Control parameter set, but slightly less dispersed (figure 6*b*). This helps to explain the slight increase in U-Richard growth rate for increases in death response width (figure 4), in that the more consistently eccentric shape of the aggregate during simulation increased the perimeter where proliferation occurs. Considering this and that all simulated parameters produced aggregates with appreciable eccentricity, the necessity of considering aggregate shape changes when modelling diffusion-limited growth is clear. Similarly, the near linear behaviour of the corresponding U-Richards fitted upper asymptote (figure 4) can be explained by that the mean of their measured eccentricity during the final 25 kMCSs was nearly the same as that of the Control parameter set, and that none of the three parameter sets during this simulation period generated a particularly low eccentricity standard deviation (i.e. their final shapes were statistically similar).

While more advanced methods are required in future work to better understand the relationships between the intracellular model parameters, aggregate shape changes, and emergent U-Richards shape parameter and growth rate, a quantitative method was devised to show how the intracellular model parameters and aggregate shape affect final size. This was accomplished by calculating a so-called ‘probability area’ of the responses of proliferation and death, which, for each response, is the area within the aggregate weighted by its corresponding probability (equation 2.4). For the *j*th response, the corresponding probability area *A*_*j*_ was calculated by integrating this mathematical characterization over an aggregate domain A,

3.1 $$A_{j}=\int_{A}\beta p_{j}^{\tau_{i}}\exp\left(-{\left(\frac{c-c_{j}^{\tau_{i}}}{\gamma_{j}^{\tau_{i}}}\right)}^{2}\right)\text{d}A,$$

where a final size can be expected to occur when the probability area of proliferation is equal to that of death (i.e. when total cell proliferation is equal to total cell death).

**Figure 7.**
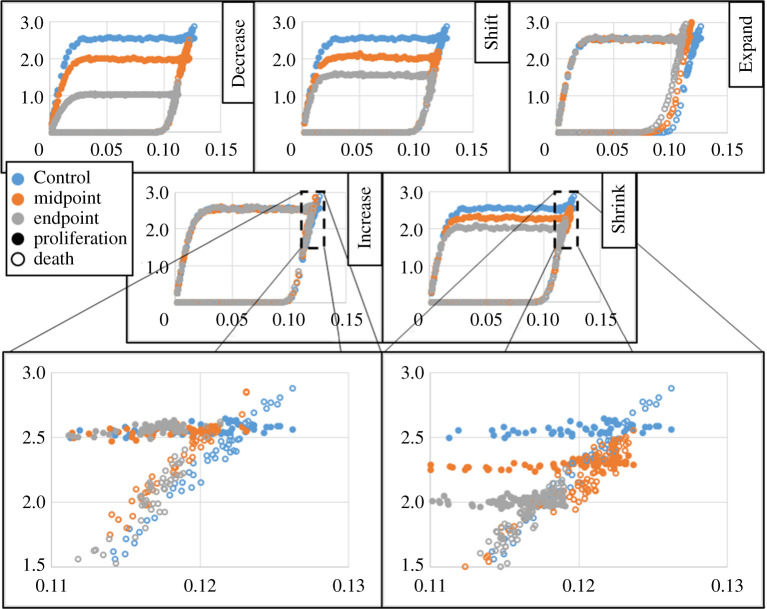
Probability-weighted areas (μm^2^, vertical) versus total aggregate area (mm^2^, horizontal). Note the constant proliferation and rightward shift in death for trials with prolonged eccentricity (figure 6).

Analysis using equation (3.1) clearly showed the effects of intracellular parameters and aggregate eccentricity on final aggregate size, as well as on the emergent growth dynamics of the simulated system, in general (figure 7). For all simulated parameter sets, an approximately constant probability area of proliferation was calculated for an aggregate size less than 0.05 mm^2^, while the measured probability area of death, after an initial, nonlinear increase, behaved as if linearly proportional to aggregate area. Furthermore, the mean magnitude of the probability area of proliferation for aggregate sizes greater 0.05 mm^2^ linearly decreased with decreasing proliferation rate (*R*^2^ = 1 when fitting this metric to varying proliferation rate in equation (2.4) with a line), linearly decreased with decreasing proliferation response width (*R*^2^ = 0.9992 when fitting this metric to varying proliferation response width with a line), and linearly decreased with increasing proliferation response target (*R*^2^ = 0.9986 when fitting this metric to varying proliferation response target with a line). Variations in death response parameters generally resulted in a leftward shift of the resulting line for probability area of death when plotted against aggregate area.

The measured constant probability area of proliferation shown in figure 7 predicts that, in a spheroid of appreciable size, the volume containing proliferating cells is approximately constant (consistently with experimental observations [45]) and, without inhibition of aggregate growth, the emergent growth curve is a straight line (consistently with assumptions used in similar modelling approaches [31,35]). This stands in stark disagreement with classical formulations of growth models, where proliferation is often considered to be proportional to aggregate surface area (where proliferation occurs [9]). In the present case, simulation results show that the model may not directly support this assumption in any appreciable way, given that the measured high eccentricity for later results from the midpoint variation in proliferation response width (figure 6) was not demonstrated in the measured probability area for proliferation.

However, this theoretical assumption of some classical growth models may be supported by simulation results, but in a rather indirect way. Like the midpoint variation in proliferation response width, the endpoint variation in death rate demonstrated a consistently high eccentricity in the later stage of simulation (figure 6). For the case of the proliferation response width, measured probability area for the response of death was expected to be unchanged among these variations, since the probability distribution of death was unchanged. And yet, for the variation with notable late eccentricity, there was also a notable upward shift in measured probability area of death (figure 7), meaning that the intersection of the probability areas of proliferation and death shifted upwards. For the case of increasing death rate, one would expect a continued upward shift in probability area of death, but this upward shift was not present for the endpoint variation, which was also highly eccentric. This can be readily explained by that the minimum distance within a spheroidal aggregate to an oxygenating interface generally decreases with increasing aggregate eccentricity. But with increasing eccentricity, the surface area of the aggregate also increases, and so some dynamic relationship between aggregate size and surface area is supported by the model. However, the model presented here redirects the emphasis on the relationships between the underlying (cellular and subcellular) mechanisms of the aggregate and the resultant emergent aggregate behaviours and properties, rather than on the relationships between the emergent behaviours and properties *per se*, which may lack a requisite level of information due to the underlying complexity. The measured linear response of final aggregate size to a linear variation in proliferation response width (figure 4) may perhaps then be a consequence of the eccentricity during simulation of the midpoint variation causing an upward deviation from an otherwise nonlinear response, and this should be investigated in future work with far more simulations using greater technological resources.

**Figure 8.**
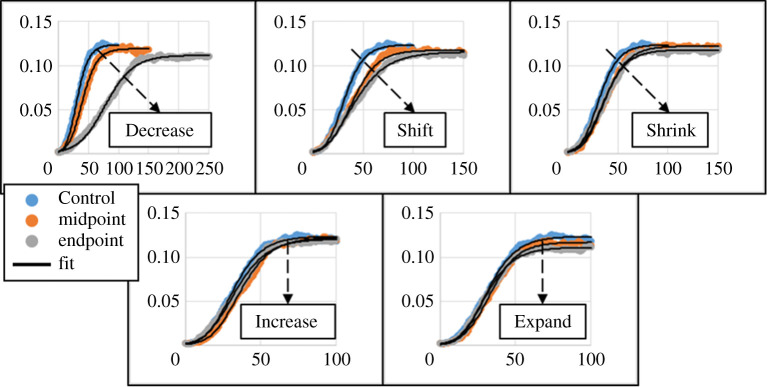
Total measured aggregate area (mm^2^, vertical) versus simulation time (kMCS, horizontal), by intracellular model parameter variation. Trajectory of dashed lines indicates inferred response of emergent curve to individual intracellular parameters from analysis.

Considering all aforementioned observations from analysis, the qualitative effects of the parameters of the intracellular response model on emergent growth dynamics as described by the unified Richards model, though perhaps obvious from the model formulation but not readily apparent from simulation results, were deduced for final size and growth rate (figure 8). The model predicts that aggregate growth rate and final size increase with increasing rate at which a cell phenotype proliferates in response to sufficient oxygen, with increasing range over which a phenotype considers oxygen concentrations to be sufficient for proliferation, and with the nearness of local oxygen to sufficient phenotype-specific levels for proliferation. Likewise, aggregate final size (but not growth rate) is predicted to decrease with increasing rate at which a phenotype responds to hypoxic conditions, and with increasing range over which a phenotype considers oxygen to be insufficient.

## 4. Discussion

Probably most obviously (and also most frustratingly), a description of the effects of the intracellular model parameters on emergent U-Richards shape parameter could not be inferred from analysis of simulation results. This is relevant since the primary goal of this work was to elucidate the discrepancy between the growth models of interest (i.e. Bertalanffy, Gompertz and logistic) for various cell phenotypes by means of the intracellular model, and the U-Richards shape parameter is clearly the means by which each model can be identified. While the cause of their discrepancy could be considered as demonstrated by the present models and method (at least for the Gompertz and logistic models, see figure 5), a quantitative understanding of how the various phenotype parameters affect the tendency of an aggregate to behave like a particular growth model would be particularly insightful (and also useful). As such, the description of the characteristic features of an emergent curve as related to the parameters of the model presented here is incomplete, and should be regarded as preliminary. The central thesis, however, is shown, in that type-specific variations of the same model framework produced curves that behaved more or less like classical growth models, as observed for type-specific responses in nature.

To generate a quantitative description of the effects of the type-specific parameters on emergent curve characteristics in future work, many more simulation results should be generated and analysed to characterize the stochastic nature of the model (especially concerning aggregate shape changes), which may then present a better means of sensitivity analysis. Such results may also allow for characterization of high sensitivity to stochastic outcomes for very low numbers of cells (as demonstrated by highly varying initial time in table 2), which was mitigated in the present work by fitting to results with 15 or more cells (like a minimum detectable size) but excludes better representative biological heterogeneity. Generating such a description would then provide a means to translate measured aggregate growth data into calibrated model parameters, which is necessary for modelling applications in medicine and development, alike (a good test of such a description, thanks in part to a question from a reviewer of this manuscript, would be the idealized inverse problem of determining the parameters of the model from simulation results using the model). This is especially true when considering that the work presented here considered an oxygen-driven system, without the additional complexity of multiple nutrients (e.g. glucose), inhibitors (e.g. lactate), extracellular matrix consistency (as in [27]), and other possible regulatory mechanisms of cell proliferation like cell shape (as in [38]). Of course, the intracellular model has a natural extension to a multivariate form of the Guassian distribution to accommodate coupled responses to multiple chemicals species. However, what is currently unclear is how to couple the Gaussian probability model with other studied regulatory mechanisms of proliferation like cell compressibility, contact inhibition and cell deformation, whether as independent or interdependent regulators. These future theoretical developments are essential for accurate predictions of spatio-temporal population dynamics in more diverse and dynamic *in vivo* microenvironments, as well as in tumour spheroids, which are also structurally heterogeneous [6].

Quantifying the mechanisms that generate each of the classical models is relevant since all classical growth models, when fitted to each trial, presented a coefficient of determination that would be considered acceptable by many standards such that one could question the need for using the unified Richards model (though it is employed here for quantifying emergence, rather than to prove its necessity). However, choosing the wrong classical model could generate wildly wrong predictions based on limited data, as is evident in figure 5 (consider if one modelled the early response of Shift 1 with the logistic model). Of course, employing more advanced metrics of goodness of fit than those used in this work might provide better insight into which classical model is best suited to describe a particular growth curve. The coefficient of determination was employed in this work for each classical model (table 2), as is commonly performed when comparing the utility of possible classical models, to show the relative goodness of fit with respect to its emergence (i.e. a better *R*^2^ for a classical model correlates with a closer corresponding U-Richards shape parameter). More importantly, the classical growth models cannot be generalized to an arbitrary environment or aggregate shape (due to their implicit representation of underlying biological complexity), while the underlying mechanisms responsible for producing the emergent behaviours that they sometimes predict can be generalized to a set of cell–environment interactions, as was observed in this work for the Gompertz model (figure 5). Such a generalized description, with explicit representations of cell–cell and cell–environment interactions like the model of this work, is then useful for predictions concerning an arbitrary environment, whether in development, medical applications, or otherwise (at the expense of additional computational cost). In this sense, the discrepancy between the classical growth models can be explained when considering them as functions in the space of all U-Richards models, where environmental conditions and a particular cell type determine the corresponding function that describes the emergent growth characteristics of the aggregate. In this same sense, knowing the cell–environment interactions from which the characteristics of the aggregate emerge is to then inform about the mapping from environmental conditions and a cell type to collective behaviour in general. This knowledge then reinforces the utility of the classical growth models, as well as promotes rational selection of which model for a particular modelling application, by providing an explicit description of the underlying biology that they implicitly represent.

To this end, the lack of growth inhibitors in the present work is probably responsible for no simulation producing a growth curve resembling the Bertalanffy model, where growth begins to slow much sooner than in the Gompertz model. In this case, a cell phenotype model could include the secretion of a growth inhibitor (either in hypoxic conditions, like lactate, or when dying, like lysis), causing the inhibition proliferation by cells in distant, well-oxygenated conditions. Such a model probably requires simulating aggregates with sizes that span multiple orders of magnitude before these effects can be readily quantified, the overall computational cost of which may require high performance computing technologies and advanced parallelization of the present model and methods. This is also the case when scaling the present model and methods to three-dimensional simulations, which is relevant because of both increased diffusion and the possibility of eccentric aggregate shape when considering a third spatial dimension. Since results demonstrated that the onset of eccentricity is stochastic (at least to some degree), a complete understanding of how phenotype-specific behaviours affect the onset of aggregate eccentricity (and thus aggregate growth) requires both three-dimensional simulations, and many trials thereof, for a particular intracellular model parameter set. This pursuit also requires variations in CPM model parameters, probably most importantly the parameters modelling adhesion, which govern the strength by which the model cells form and maintain contact interfaces. These considerations for future development of the model, whether theoretical or computational, are particularly relevant when considering the potential for performing parameter estimation when fitting to experimental data. Such a combined computational and experimental work could provide new opportunities for applying *in vitro* observations to simulations of *in vivo* scenarios, and would require rigorous evaluation of predictive capabilities.

## 5. Conclusion

In this work, a computational method was developed and employed to demonstrate that differing models of growth dynamics emerge from a phenotype-specific, statistical description of intracellular processes in response to local chemical conditions. This was accomplished by representing the subcellular mechanisms responsible for cell proliferation and death in response to local oxygen conditions as Gaussian probability distributions, which were incorporated into a hybrid cellular Potts model, simulated for diffusion-limited aggregate growth in idealized environmental conditions, and analysed according to the unified Richards model generalization of popular growth models. System stochasticity and reliability of fits using the unified Richards model were interrogated by simulating multiple trials of select sets of model parameters, and model parameters describing phenotype-specific behaviours were varied to better understand and characterize their effects on the emergent dynamics of aggregates.

Analysis of simulation morphologies showed the significance of potential aggregate shape changes concerning characterization and prediction of aggregate growth dynamics. Analysis of internal aggregate conditions showed that the intracellular model well predicts experimental observations concerning regions of cell proliferation within aggregates, which have been assumed in comparable computational approaches. Evidence was provided to explain assumed dynamic relationships employed in traditional mathematical formulations describing aggregate growth.

## Supplementary Material

Supplementary Materials

Reviewer comments

## Data accessibility

All simulation scripts used and results data presented in this work are available as electronic supplementary material.
