# Supplementary material for: Unification of aggregate growth models by emergence from cellular and intracellular mechanisms
Source: R Soc Open Sci. 2020 Aug 12;7(8):192148. doi: 10.1098/rsos.192148 (PMC7481681; doi:10.1098/rsos.192148)
Supplement: Reviewer comments [file rsos192148_review_history.pdf]

THE ROYAL SOCIETY
PUBLISHING

ROYAL SOCIETY
OPEN SCIENCE

Unification of aggregate growth models by emergence from cellular and intracellular mechanisms

T.J. Sego, James A. Glazier and Andres Tovar

Article citation details
R. Soc. open sci. **7**: 192148.
http://dx.doi.org/10.1098/rsos.192148

Review timeline

Original submission:	7 December 2019	
Revised submission:	27 May 2020	
Final acceptance:	3 July 2020	

Note: Reports are unedited and appear as submitted by the referee. The review history appears in chronological order.

Note: This manuscript was transferred from another Royal Society journal with peer review.

Review History

RSOS-192148.R0 (Original submission)

Review form: Reviewer 1

Is the manuscript scientifically sound in its present form?
No

Are the interpretations and conclusions justified by the results?
No

Is the language acceptable?
Yes

Do you have any ethical concerns with this paper?
No

Have you any concerns about statistical analyses in this paper?
Yes

Recommendation?
Major revision is needed (please make suggestions in comments)

Reports © 2020 The Reviewers; Decision Letters © 2020 The Reviewers and Editors; Responses © 2020 The Reviewers, Editors and Authors. Published by the Royal Society under the terms of the Creative Commons Attribution License http://creativecommons.org/licenses/by/4.0/, which permits unrestricted use, provided the original author and source are credited

Comments to the Author(s)
This paper asks a very interesting and important questions. Why do some classical models work better for some cancers, but not for others, and can aggregate growth dynamics on the cellular level explain the different classical model representations? The authors use the Cellular Potts Model with various parameters to simulate different growth dynamics and then fit different growth laws. The CPM explained disagreement of in silico diffusion-limited growth as emergent behaviors from the same model.

My major concern is that this important work is motivated by a different thought of train. The authors lead with "Currently, no classical model provides a general mathematical representation of growth for any cell type and environment. This discrepancy diminishes their range of applications, and a general modeling framework is needed". I wholeheartedly disagree with these statements. First, I don't think that a general model is needed to represent any and all cell types and environments. Many cell types and environments are dictated by different biology and, thus, the models should be different. There is not general model for a general tissue (or disease) and a general question. Each question and each biological entity is different, and so could (and probably should) the models.

Second, I don't think the authors solve this "problem" in this contribution. The CPM is not shown to fit and predict any data beyond data generated with the CPM. While the contribution is very interesting, its motivation and framing limits the enthusiasm.

Different statements are taken out of context. For example, "growth data from mammography screenings showed that in vivo tumor growth can be unbounded" (page 2). Nothing in nature (and not in the female breast) can be unbounded. Early tumor growth can sure appear to be (as it will be on the lower (V/K) in the logistic and Gompertz models, but it's not unbounded. The data in [13] represents an estimated average, with fixed carrying capacities from different estimation studies.

When comparing model fits to data, the Unified Richards model fits the data with best R2. The authors acknowledge that this is due to additional degree(s) of freedom over classical models, but fail to use Akaike or Bayesian Information criteria to balance number of parameters with goodness of fit. The difference in R2 is less than 1% which does not appear to justify additional degrees of freedom in the UR model.
The fits for the UR model include initial time variations from 6,000-16,000 monte carlo steps. With the initial time being known, this variation allows for tremendous fitting freedom. What happens if the time is being forced fixed?

When evaluating model predictions, the authors appear to use data generated by the CPM. Predictive power though does not seem to be rigorously evaluated beyond goodness of fit. Receiver operating characteristics and AUC analysis may be better suited. The generated data, however, shows insignificant variation compared to intra and inter-experimental heterogeneity in biology.

The authors state that R2 for all classical model fits correlate with UR shape parameter. This is not shown, and unclear.

Review form: Reviewer 2

Is the manuscript scientifically sound in its present form?
Yes

Are the interpretations and conclusions justified by the results?
Yes

Is the language acceptable?
Yes

Do you have any ethical concerns with this paper?
No

Have you any concerns about statistical analyses in this paper?
No

Recommendation?
Accept as is

Comments to the Author(s)
The authors did a commendable job responding to very detailed criticism from three referees. The new submission of this paper addresses all three reviewers adequately.

In particular, I would like to point out that this version of the paper is more accessible to readers from outside the core field, while retaining critical technical descriptions of the model.

This is a welcomed contribution to "Open Science".

Decision letter (RSOS-192148.R0)

We hope you are keeping well at this difficult and unusual time. We continue to value your support of the journal in these challenging circumstances. If Royal Society Open Science can assist you at all, please don't hesitate to let us know at the email address below.

Dear Dr Sego,

The editors assigned to your paper ("Unification of Aggregate Growth Models by Emergence from Cellular and Intracellular Mechanisms") have now received comments from reviewers. We would like you to revise your paper in accordance with the referee and Associate Editor suggestions which can be found below (not including confidential reports to the Editor). Please note this decision does not guarantee eventual acceptance.

Please submit a copy of your revised paper before 28-May-2020. Please note that the revision deadline will expire at 00.00am on this date. If we do not hear from you within this time then it will be assumed that the paper has been withdrawn. In exceptional circumstances, extensions may be possible if agreed with the Editorial Office in advance. We do not allow multiple rounds of revision so we urge you to make every effort to fully address all of the comments at this stage. If deemed necessary by the Editors, your manuscript will be sent back to one or more of the original reviewers for assessment. If the original reviewers are not available, we may invite new reviewers.

To revise your manuscript, log into http://mc.manuscriptcentral.com/rsos and enter your Author Centre, where you will find your manuscript title listed under "Manuscripts with Decisions." Under "Actions," click on "Create a Revision." Your manuscript number has been appended to denote a revision. Revise your manuscript and upload a new version through your Author Centre.

When submitting your revised manuscript, you must respond to the comments made by the referees and upload a file "Response to Referees" in "Section 6 - File Upload". Please use this to document how you have responded to the comments, and the adjustments you have made. In order to expedite the processing of the revised manuscript, please be as specific as possible in your response.

In addition to addressing all of the reviewers' and editor's comments please also ensure that your revised manuscript contains the following sections as appropriate before the reference list:

• Ethics statement (if applicable)
If your study uses humans or animals please include details of the ethical approval received, including the name of the committee that granted approval. For human studies please also detail whether informed consent was obtained. For field studies on animals please include details of all permissions, licences and/or approvals granted to carry out the fieldwork.

• Data accessibility
It is a condition of publication that all supporting data are made available either as supplementary information or preferably in a suitable permanent repository. The data accessibility section should state where the article's supporting data can be accessed. This section should also include details, where possible of where to access other relevant research materials such as statistical tools, protocols, software etc can be accessed. If the data have been deposited in an external repository this section should list the database, accession number and link to the DOI for all data from the article that have been made publicly available. Data sets that have been deposited in an external repository and have a DOI should also be appropriately cited in the manuscript and included in the reference list.

If you wish to submit your supporting data or code to Dryad (http://datadryad.org/), or modify your current submission to dryad, please use the following link:
http://datadryad.org/submit?journalID=RSOS&manu=RSOS-192148

• Competing interests
Please declare any financial or non-financial competing interests, or state that you have no competing interests.

• Authors’ contributions
All submissions, other than those with a single author, must include an Authors’ Contributions section which individually lists the specific contribution of each author. The list of Authors should meet all of the following criteria; 1) substantial contributions to conception and design, or acquisition of data, or analysis and interpretation of data; 2) drafting the article or revising it critically for important intellectual content; and 3) final approval of the version to be published.

All contributors who do not meet all of these criteria should be included in the acknowledgements.

We suggest the following format:
AB carried out the molecular lab work, participated in data analysis, carried out sequence alignments, participated in the design of the study and drafted the manuscript; CD carried out the statistical analyses; EF collected field data; GH conceived of the study, designed the study, coordinated the study and helped draft the manuscript. All authors gave final approval for publication.

• Acknowledgements
Please acknowledge anyone who contributed to the study but did not meet the authorship criteria.

• Funding statement
Please list the source of funding for each author.

Once again, thank you for submitting your manuscript to Royal Society Open Science and I look forward to receiving your revision. If you have any questions at all, please do not hesitate to get in touch.

Kind regards,
Andrew Dunn
Royal Society Open Science Editorial Office
Royal Society Open Science
openscience@royalsociety.org

on behalf of Dr Marco Viceconti (Associate Editor) and Pietro Cicuta (Subject Editor)
openscience@royalsociety.org

Associate Editor's comments (Dr Marco Viceconti):

While we agree with the second reviewer that the paper has been improving considerably through the review process, the issue raised by the first reviewer is important, and we think it requires an additional review round.

Comments to Author:

Reviewers' Comments to Author:
Reviewer: 1

Comments to the Author(s)
This paper asks a very interesting and important questions. Why do some classical models work better for some cancers, but not for others, and can aggregate growth dynamics on the cellular level explain the different classical model representations? The authors use the Cellular Potts Model with various parameters to simulate different growth dynamics and then fit different growth laws. The CPM explained disagreement of in silico diffusion-limited growth as emergent behaviors from the same model.

My major concern is that this important work is motivated by a different thought of train. The authors lead with "Currently, no classical model provides a general mathematical representation of growth for any cell type and environment. This discrepancy diminishes their range of applications, and a general modeling framework is needed". I wholeheartedly disagree with these statements. First, I don't think that a general model is needed to represent any and all cell types and environments. Many cell types and environments are dictated by different biology and, thus, the models should be different. There is not general model for a general tissue (or disease) and a general question. Each question and each biological entity is different, and so could (and probably should) the models.

Second, I don't think the authors solve this "problem" in this contribution. The CPM is not shown to fit and predict any data beyond data generated with the CPM. While the contribution is very interesting, its motivation and framing limits the enthusiasm.

Different statements are taken out of context. For example, "growth data from mammography screenings showed that in vivo tumor growth can be unbounded" (page 2). Nothing in nature (and not in the female breast) can be unbounded. Early tumor growth can sure appear to be (as it will be on the lower (V/K) in the logistic and Gompertz models, but it's not unbounded. The data

in [13] represents an estimated average, with fixed carrying capacities from different estimation studies.

When comparing model fits to data, the Unified Richards model fits the data with best R2. The authors acknowledge that this is due to additional degree(s) of freedom over classical models, but fail to use Akaike or Bayesian Information criteria to balance number of parameters with goodness of fit. The difference in R2 is less than 1% which does not appear to justify additional degrees of freedom in the UR model.
The fits for the UR model include initial time variations from 6,000-16,000 monte carlo steps. With the initial time being known, this variation allows for tremendous fitting freedom. What happens if the time is being forced fixed?

When evaluating model predictions, the authors appear to use data generated by the CPM. Predictive power though does not seem to be rigorously evaluated beyond goodness of fit. Receiver operating characteristics and AUC analysis may be better suited. The generated data, however, shows insignificant variation compared to intra and inter-experimental heterogeneity in biology.

The authors state that R2 for all classical model fits correlate with UR shape parameter. This is not shown, and unclear.

Reviewer: 2

Comments to the Author(s)
The authors did a commendable job responding to very detailed criticism from three referees. The new submission of this paper addresses all three reviewers adequately.

In particular, I would like to point out that this version of the paper is more accessible to readers from outside the core field, while retaining critical technical descriptions of the model.

This is a welcomed contribution to “Open Science”.

Author's Response to Decision Letter for (RSOS-192148.R0)

See Appendix A.

RSOS-192148.R1 (Revision)

Review form: Reviewer 1

Is the manuscript scientifically sound in its present form?
Yes

Are the interpretations and conclusions justified by the results?
Yes

Is the language acceptable?
Yes

Do you have any ethical concerns with this paper?
No

Have you any concerns about statistical analyses in this paper?
No

Recommendation?
Accept as is

Comments to the Author(s)
The authors have responded to my initial concerns. I have no further recommendations for improvement of the revised manuscript

Decision letter (RSOS-192148.R1)

We hope you are keeping well at this difficult and unusual time. We continue to value your support of the journal in these challenging circumstances. If Royal Society Open Science can assist you at all, please don't hesitate to let us know at the email address below.

Dear Dr Sego,

It is a pleasure to accept your manuscript entitled "Unification of Aggregate Growth Models by Emergence from Cellular and Intracellular Mechanisms" in its current form for publication in Royal Society Open Science. The comments of the reviewer(s) who reviewed your manuscript are included at the foot of this letter.

Please ensure that you send to the editorial office an editable version of your accepted manuscript, and individual files for each figure and table included in your manuscript. You can send these in a zip folder if more convenient. Failure to provide these files may delay the processing of your proof. You may disregard this request if you have already provided these files to the editorial office.

You can expect to receive a proof of your article in the near future. Please contact the editorial office (openscience_proofs@royalsociety.org) and the production office (openscience@royalsociety.org) to let us know if you are likely to be away from e-mail contact -- if you are going to be away, please nominate a co-author (if available) to manage the proofing process, and ensure they are copied into your email to the journal.

Due to rapid publication and an extremely tight schedule, if comments are not received, your paper may experience a delay in publication.

Please see the Royal Society Publishing guidance on how you may share your accepted author manuscript at https://royalsociety.org/journals/ethics-policies/media-embargo/.

Thank you for your fine contribution. On behalf of the Editors of Royal Society Open Science, we look forward to your continued contributions to the Journal.

Kind regards,
Anita Kristiansen
Editorial Coordinator

Royal Society Open Science
openscience@royalsociety.org

on behalf of Pietro Cicuta (Subject Editor)
openscience@royalsociety.org

Associate Editor Comments to Author:

Comments to the Author:

The paper is now ready for acceptance - congratulations.

Reviewer comments to Author:
Reviewer: 1

Comments to the Author(s)

The authors have responded to my initial concerns. I have no further recommendations for improvement of the revised manuscript

Follow Royal Society Publishing on Twitter: @RSocPublishing
Follow Royal Society Publishing on Facebook:
https://www.facebook.com/RoyalSocietyPublishing.FanPage/
Read Royal Society Publishing's blog: https://blogs.royalsociety.org/publishing/

Appendix A

Reviewer #1

This paper asks a very interesting and important questions. Why do some classical models work better for some cancers, but not for others, and can aggregate growth dynamics on the cellular level explain the different classical model representations? The authors use the Cellular Potts Model with various parameters to simulate different growth dynamics and then fit different growth laws. The CPM explained disagreement of in silico diffusion-limited growth as emergent behaviors from the same model.

R1.1 *My major concern is that this important work is motivated by a different thought of train. The authors lead with "Currently, no classical model provides a general mathematical representation of growth for any cell type and environment. This discrepancy diminishes their range of applications, and a general modeling framework is needed". I wholeheartedly disagree with these statements. First, I don't think that a general model is needed to represent any and all cell types and environments. Many cell types and environments are dictated by different biology and, thus, the models should be different. There is not general model for a general tissue (or disease) and a general question. Each question and each biological entity is different, and so could (and probably should) the models.*

Response: For descriptions that use ordinary differential equations (ODEs) like the classical growth models, we completely agree that each tissue is different and probably requires a particular mathematical form. As we have shown in the work (and one would expect), the particular form is determined by the cellular/subcellular factors, as well as the particular biological milieu, causing the emergent behaviors and properties from which the growth curves emerge. Fundamentally, this is really not much different from particularizing the framework presented in the manuscript (*i.e.*, for a given tissue, find the appropriate ODE vs. find the appropriate model coefficients). Really, what's meant by "a general mathematical representation" and "diminishes their range of application" is that the ODE forms do not contain an explicit representation of phenotypic diversity and spatial information in the dynamical forms. We could have better claimed that the range of applications of the ODEs is limited by the level of biological complexity that is implicitly contained in the mathematical form (*e.g.*, aggregate shape). Of course, one could just as well argue that the range of applications of the explicit, cellular-level forms is limited by the computational resources required to use them! It would be better to claim that relating the tissue-level ODEs to explicit representations of cell, phenotype, and spatiality augments the insights that they provide. Furthermore, explaining their disagreement in terms of these explicit, cellular-level dynamical forms improves their overall validity by providing a means by which their disagreement can be explained. Plainly stated, the cause of their disagreement can be explained through the cellular-level system presented in this work, which the authors feel advances the knowledge to this field of research. We have revised the manuscript to reflect these sentiments, which can be found in the abstract and third paragraph of Discussion, and thank the reviewer for helping us better clarify the relationship between the classical

models and ours.

R1.2 *Second, I don't think the authors solve this "problem" in this contribution. The CPM is not shown to fit and predict any data beyond data generated with the CPM. While the contribution is very interesting, its motivation and framing limits the enthusiasm.*

Response: We share the reviewer's appreciation for model calibration to experimental data. However, the work was motivated by theoretical aspects of modeling growth dynamics, rather than calibrating parameters to a particular experimental data set. Plainly stated, the scope of the present work was based on the following premise: it is well known that different tissues follow different tissue-level ordinary differential equations; however, it is not well known why a particular tissue follows a particular ordinary differential equation. For the present work, we felt it sufficient to operate on these observations, which we have tried very hard to establish for the reader in the introduction, and that providing fits to specific *in vitro* data sets would likely not contribute much more to the scope of the present work compared to the computational cost and additional documentation of doing so. However, this is an excellent point of future work that motivated the present work in the first place, specifically on developing computational workflows to do parameter estimation from data sets to characterize phenotype-specific responses. This is especially important for applications in predictive capabilities of *in vivo* scenarios, where we translate knowledge gained from *in vitro* parameter estimation into useful simulations of events of which time-course measurements are very difficult to make. We have added discussion of developing such workflows in the final paragraph of Discussion.

R1.3 *Different statements are taken out of context. For example, "growth data from mammography screenings showed that in vivo tumor growth can be unbounded" (page 2). Nothing in nature (and not in the female breast) can be unbounded. Early tumor growth can sure appear to be (as it will be on the lower (V/K) in the logistic and Gompertz models, but it's not unbounded. The data in [13] represents an estimated average, with fixed carrying capacities from different estimation studies.*

Response: We thank the reviewer for pointing out this issue, which is due to unclear wording in the text. As the reviewer remarks, nothing in nature is unbounded, and the statement referenced by the reviewer was intended to point to an instance in the literature where the best-fit classical model predicted unbounded growth (quite a difference, indeed!). We have revised the manuscript to make this clear, which can be found in the third paragraph of Background.

R1.4 *When comparing model fits to data, the Unified Richards model fits the data with best R2. The authors acknowledge that this is due to additional degree(s) of freedom over classical models, but fail to use Akaike or Bayesian Information criteria to balance*

number of parameters with goodness of fit.

Response: We appreciate the reviewer's attention to judging the applicability of the classical growth models to a particular dataset using appropriate, comparative methods. We use R^2 since this metric is commonly used to judge the usefulness of a particular ODE to describe a dataset, and to quantitatively show characteristics related to emergence with respect to variations in parameters (rather than to choose a classical model). As the reviewer points out, applying these additional metrics to compare various fits and the U-Richards model could provide further insights into which model to use for tissue-level characterization. However, the present work is completely agnostic about which classical model to use, and is entirely focused on providing insights into why they disagree. We have added content to the third paragraph of Discussion that clarifies the purpose of using the coefficient of determination, as well as an acknowledgment of using better methods when selecting a classical model for a dataset.

R1.5 *The difference in R2 is less than 1% which does not appear to justify additional degrees of freedom in the UR model.*

Response: We agree with the reviewer that the referenced R^2 results would not merit using the Unified-Richards model. However, the present work does not advocate for a particular growth model (as discussed in R1.4), neither does it advocate for the usage of the Unified-Richards model. Rather, we use the Unified-Richards model (specifically, its shape parameter) to characterize emergence. We have added content in the last paragraph of Background and third paragraph of Discussion to clarify the purpose of the Unified-Richards model in our methods.

R1.6 *The fits for the UR model include initial time variations from 6,000-16,000 monte carlo steps. With the initial time being known, this variation allows for tremendous fitting freedom. What happens if the time is being forced fixed?*

Response: Indeed, the fits are different, and much noisier (as described in the first paragraph of Resuls). For a very small number of cells, the effects of stochasticity are non-negligible, which introduces a significant amount of noise to the analysis method. However, this freedom of choice in "time zero" is not outside of scope of fitting curves to growth data (since "time zero" *in vivo* corresponds to "time detected"). We would like to note that the stochasticity and cellular resolution of the model presented in the manuscript provides a more detailed accounting of this aspect of biological complexity than its higher-level, ODE counterparts. But these considerations speak more to the difficulty in predicting asymptotic growth from early data in general, which is not unique to the model of this work, and we did not feel that including a detailed accounting of them particularly insightful with respect to the scope of the work. However, characterizing these effects of early sensitivity to stochasticity is likely possible

through computational approaches using the model presented in the manuscript. We have included discussion of this in the second paragraph of Discussion, including variations in initial time, sensitivity of small populations to stochastic processes, and the comparison of our filtering to a minimum detectable size.

R1.7 *When evaluating model predictions, the authors appear to use data generated by the CPM. Predictive power though does not seem to be rigorously evaluated beyond goodness of fit. Receiver operating characteristics and AUC analysis may be better suited. The generated data, however, shows insignificant variation compared to intra and inter-experimental heterogeneity in biology.*

Response: We do use data generated by the framework that includes the CPM to show the hypothesis of our work, since the scope of the work was to show emergent behavior, rather than to calibrate to, or predict, a particular experimental dataset (as described in R1.2). We do this based on points described in Background, specifically that the traditional growth models have case-specific utility, and that the U-Richards model generalizes the growth models of interest. However, rigorous evaluation of the predictive capabilities when pursuing such endeavors is necessary, which we have included in the final paragraph of Discussion when addressing R1.2. Along augment current content about limited heterogeneity of the generated data (*e.g.*, the final paragraph of Discussion), we have added to the second paragraph of Discussion when addressing R1.5 that our filtering of very early results removes heterogeneity from the fitted data (as intended for the scope of the present work).

R1.8 *The authors state that R2 for all classical model fits correlate with UR shape parameter. This is not shown, and unclear.*

Response: We thank the author for pointing out that this requires a clearer description. Along with further clarifying the intent of using the Unified-Richards model when addressing R1.4 and R1.5, we have also added content to the fifth paragraph of Results to bring attention to that shape parameters near a Unified-Richards model description (*e.g.*, $S \to 1$ for Gompertz-like curves) correlate with R^2 nearer to 1.

Reviewer #2

The authors did a commendable job responding to very detailed criticism from three referees. The new submission of this paper addresses all three reviewers adequately.

In particular, I would like to point out that this version of the paper is more accessible to readers from outside the core field, while retaining critical technical descriptions of the model.

This is a welcomed contribution to "Open Science".

Response: The authors would like to thank the reviewer for their time reviewing the manuscript and previous feedback. We appreciate the positive feedback about both the accessibility and technical description of the manuscript.
